# Brazilian National Anthem presenting as musical hallucination: A case report with 9-year follow-up

**DOI:** 10.1590/S1980-5764-2016DN1003013

**Published:** 2016

**Authors:** José Eduardo Martinelli, Juliana Francisca Cecato, Ivan Aprahamian

**Affiliations:** 1 Department of Internal Medicine, Faculty of Medicine of Jundiaí, São Paulo, Brazil.

**Keywords:** elderly, musical hallucination, differential diagnosis, treatment

## Abstract

Musical hallucination is a type of complex auditory hallucination. Possible etiologies are deafness, psychiatric disorders such as schizophrenia, major depression, use of medication and stress, besides neurologic diseases including epilepsy, stroke and cancer. Uncommon etiologies encompass infectious diseases, metabolic disorders, and sensory deprivation. Although musical hallucinations have a major impact on patients' lives, they have been undervalued and understudied in the literature. We report a case of a 79-year-old woman with musical hallucination (hearing a sung National anthem) without cognitive impairment or hearing loss. The patient had preserved insight of her complaint and responded well to neuroleptics.

## RESUMO

A alucinação musical é o um tipo de alucinação auditiva complexa. As etiologias possíveis são a perda auditiva, transtornos psiquiátricos tais como a esquizofrenia, depressão maior, uso de medicações e estresse, condições neurológicas como a epilepsia, acidente vascular encefálico e neoplasias. Etiologias menos frequentes englobam doenças infecciosas, metabólicas e endócrinas e privação sensorial. Apesar das alucinações musicais causarem grandes repercussões na vida dos pacientes sempre foram pouco valorizadas e estudadas na literatura. Relatamos o caso de uma senhora de 79 anos com alucinação musical (ouvia o hino nacional cantado), sem déficit cognitivo ou perda auditiva. A paciente tinha *insight* de seu problema e respondeu bem ao tratamento com neurolépticos.

## INTRODUCTION

Hallucinations are experiences similar to real perception yet with no external stimulus. They are vivid and bright, with all the strength and impact of normal perceptions, and are not under voluntary control. They can occur in any sensory modality, although auditory hallucinations are the most common in schizophrenia and related disorders.^1^

Musical hallucination (MH) is a complex auditory hallucination type described as hearing musical tones, rhythms, harmonies and melodies without a corresponding external auditory stimuli in patients that are not necessarily affected by a psychopathological disorder. MH are often associated with serious hearing problems, although other causes exist. Some patients never discover a definite cause. These hallucinations can be continuous or intermittent and occur in clear conscience and with preserved insight.^2^

MH are predominantly found in older women with progressive hearing loss secondary to general diseases or specific ear injuries. They also occur in neurological disorders (mainly focal brain lesions, epilepsy originating from dementia and temporal lobes), psychiatric disorders, especially depression and intoxication, as a sole cause or in combination. MH have been described following the use of tricyclic antidepressants (clomipramine, imipramine), salicylates, benzodiazepines, pentoxifylline, propranolol, carbamazepine, phenytoin, alcohol abuse and general anesthesia.^3^

MH are underdiagnosed and undervalued by health professionals, despite the fact that these symptoms can significantly impair patients' quality of life. A small number of studies have been conducted to elucidate the pathophysiological process of MH, while few studies report successful treatment of these symptoms.^4^

MH were first described in the literature in the nineteenth century by Baillarger (1846) and Griessinger (1867).^5^ During the same period, the celebrated composer R. Schumann reported MH experience in the context of a psychotic disorder (bipolar disorder?, neurosyphilis?), which he subsequently incorporated into his musical composition.^6^ The first report of MH associated with hearing loss was published by W.S. Colman in 1894. Since then, sporadic reports have emerged and MH are now considered rare. MH were the focus of systematic studies during the 1970s in an attempt to elucidate its pathogenesis. It is estimated that about 2% of the elderly with hearing loss also have MH and it is probably the most frequent non-psychotic hallucination.^7^

Incidence of MH is estimated as one new case per 10,000 persons per year with psychiatric illness over the age of 65 years.^8^ Prevalence of MH is highly heterogeneous, ranging from 0.86% to 2.5% in an elderly population with hipoacusis.^9^ It is a rare phenomenon in general hospitals. However, in patients with psychiatric illness, MH rates as high as 20% have been reported, more frequently observed with obsessive-compulsive disorder, major depression and schizophrenia.^10^

The most accepted pathogenesis for MH associated with hearing loss and sensory deprivation (caused by a cochlear injury or information interruption at the pons or midbrain) is disinhibition of auditory memory circuits. Based on this prerogative, some authors argue that MH can be a form of auditory Charles-Bonnet syndrome, causing abnormal activity in music processing cortical modular segments.^11^ The neuropsychological basis of MH is not fully established, but also suggests the involvement of central auditory processing mechanisms.^12^ Musical auditory processing involves various levels of interconnected brain subsystems. While the recognition of elementary sounds is made by the primary auditory cortex, the recognition of musical characteristics such as musical notes, melody, rhythm, and metric occurs in secondary and tertiary association centers, which, in turn, are influenced by regions connected to memory and emotion circuits. There is evidence that, in the case of MH, an excitatory mechanism in the superior temporal cortex, as in epilepsy, is responsible.^12^ In cases of MH without hearing loss, there may be a possible disconnection of afferent or cortical networks due to lesion or physiological aging and associated common comorbidities, such as systemic hypertension and diabetes.^13^

We report the case of an elderly woman with preserved cognition and insight of her disorder (heard the National anthem sung 24 hours a day) and with no psychiatric disorders.

## CASE REPORT

A 79-year-old woman with 11 years of formal education sought the geriatric outpatient facility of the Faculty of Medicine of Jundiaí complaining of hearing the National Anthem sung 24 hours a day. During the daytime she took to turning the radio or television up to a high volume to avoid hearing the anthem. At night the music became more intense making it difficult to sleep. After 3 months of persistence of the hallucination she became very anxious, stressed and irritated. She had preserved insight of her problem and reported no other types of hallucination, depressive symptoms, memory or cognitive complaints. She had used bromazepam 3 mgpreviously for a sleep disorder and began using it again after this period. She slept for around 4 hours per night. Physical, psychiatric, and neurological examinations were normal. 

Her medical background consisted of systemic hypertension, dyslipidemia, sporadic vertigo diagnosed as labyrinthitis, and coronary disease. The patient underwent coronary artery bypass surgery 2 years earlier. She also reported regular use of atorvastatin 20 mg, metoprolol 50mg, enalapril with hydrochlorothiazide (20/12.5 mg), buffered aspirin 100mg, and nicergoline 30mg. There was no family history of psychiatric illness.

Despite an absence of hearing complaints, the patient underwent an audiometry exam. The audiometry test was normal. Biochemical tests (urea, creatinine, blood glucose, complete blood count, cholesterol profile, CRP and TSH) and magnetic resonance of the brain were normal. Complementary and neuropsychological evaluation consisted of the Cambridge Examination for Mental Disorders of the Elderly (CAMDEX) and a cognitive battery comprising the Cambridge Cognitive Examination (CAMCOG),^14^ the Mini-Mental State Examination (MMSE),^15^ ^,^ ^16^ the Clock Drawing Test (CDT) using Mendez^17^ and Shulman^18^ scales, the short Geriatric Depression Scale (GDS),^19^ and Pfeffer's Functional Activities Questionnaire (FAQ.)^20^ All of these tests were normal (Table 1).

**Table 1:** Results on instruments evaluating cognitive and depressive symptoms, and functional performance.

Instrument	Subject score	Cut-off point
MMSE	29	25
CAMCOG	92	80
Mendez CDT	20	18
Shulman CDT	5	3
FAQ	0	<5
GDS-short	6	<5

MMSE: Mini-Mental State Examination; CAMOCG: Cambridge Examination for Mental Disorders of the Elderly; CDT: Clock Drawing Test using Mendez and Shulman scales; GDS-short: 15-item Geriatric Depression Scale; FAQ: Pfeffer's Functional Activities Questionnaire.

She was treated with risperidone 0.5 mg daily for 6 months and showed complete remission. At 6 months after withdrawal of the medication she exhibited no further MH. She has been followed thereafter with no relapses for 9 years.

## DISCUSSION 

What calls attention in this case is that the patient sought a geriatric clinic with the chief complaint of hearing the National anthem sung, uninterrupted, 24 hours a day, for more than three months. We have conducted a systematic search on PubMed (MeSH terms "musical hallucinations" and "elderly", 25 case reports/case series) and LILACS (advanced search with terms "musical", "hallucinations" and "elderly", no reports) in a bid to identify similar case reports. All 25 studies reported on PubMed involved elderly with musical hallucinations associated with adverse reactions to medications, dementia or cognitive decline, deafness or hearing impairment, and cerebrovascular disease. 

In most reported cases, MH is part of other psychiatric disorders as opposed to the chief complaint for which the patient seeks medical attention. Our patient repeatedly asked her husband where the song was coming from, despite preserved insight. Griffiths et al. reported that people with MH initially believed that music was actually playing somewhere else (especially at the neighbors).^21^ In some subjects, MH manifests abruptly and are initially interpreted as coming from outside. Generally, the content of MH are melodies and songs familiar to patients who often have not heard them since childhood. Religious songs are prevalent in many cases. Many of these songs are seasonally related (June festivals, Christmas, Carnival). MH are also a reflection of individual music collection.^2^ ^,^ ^8^ There are no reports of spontaneous remission of MH.^22^

According to its origin, MH can be classified as functional or organic. The functional type is associated with psychopathological disorders with no apparent physical damage to the brain or presence of hearing loss, such as schizophrenia, depression and obsessive-compulsive disorder.^23^ Boza established six categories of non-psychiatric MH, two of which may fit our case, i.e. psychological and environmental origins.^24^ Organic MH occur mostly in patients with acquired severe hearing loss, with other causes including cerebrovascular lesions, brain tumors, among others.^4^

In the case reported, we did not observe the presence of affective symptoms or other psychotic manifestations. She presented with a high level of emotional stress and anxiety. She has a son with Down syndrome, age 50, whose care left her tired; her daughter-in-law did not allow her to see the grandchildren owing to family rifts. Previously, at 45 years old, she had major depression and after remission presented no further episodes. After onset of MH, her quality of life had declined and she had become more distressed, but without clinical symptoms consistent with major depression. She scored 6 on the Geriatric Depression Scale, which could classify her as having a mild depression, but did not score on the CAMDEX depression scale. Santos et al. reported a series of 8 subjects from an ENT clinic with MH and variable degrees of depression.^25^ We assumed that her MH was due to psychological stress. 

The patient showed no cognitive impairment during the 9-year follow-up or abnormality on brain resonance imaging (MRI). She also did not present with hearing loss and her audiometry was normal. Kasei et al. had also described a woman with normal audiometry and MRI who presented abrupt MH in the form of well-known songs.^21^ SPECT imaging in the presence and absence of MH showed changes consistent with excitation specific to areas of the auditory association cortex and an increase in blood flow in the right superior temporal gyrus during MH.^21^

## CONCLUSION

The pathogenesis and neuropsychological basis of MH is not fully established, but might involve central auditory processing mechanisms. MH are mainly associated with hearing loss, psychiatric and neurological disorders (especially after brain damage or epilepsy originating from the temporal lobes) and intoxication, as a single cause or in combination. However, MH are also seen in healthy subjects. This phenomenon tends to be underdiagnosed if not actively explored. In the literature, there are few reports of healthy patients with MH, especially without hearing or cognitive impairments. 

## References

[1] American Psychiatric Association (2014). Diagnostic and Statistical Manual of Mental Disorders - DSM-5.

[2] Zabalza-Estévez RJ (2014). Musical hallucinations perpetual music. Rev Neurol.

[3] Klut C, Xavier S, Graça J, Cardoso G (2011). Alucinações musicais e esquizofrenia a propósito de um caso
clínico. Psilogos.

[4] Sanchez TG, Rocha SCM, Knobel KAB, Kii MA, Santos RMR, Pereira CB (2011). Musical hallucination associated with hearing loss. Arq Neuro-Psiquiatr.

[5] Berrios GE (1990). Musical hallucinations A historical and clinical study. Br J Psychiatry.

[6] Stewart L, Von Kriegstein K, Warren JD, Griffiths TD (2006). Music and the brain disorders of musical listening. Brain.

[7] Magalhães RS, Silva AA, Salaroli AF, Pachoal JR (2008). Alucinações musicais em portadores de perda auditiva. Rev Bras Otorrinolaringoloiga.

[8] Warner N, Aziz V (2005). Hymns and arias musical hallucinations in older people in
Wales. Int J Geriatr Psychiatry.

[9] Evers S (2006). Musical hallucinations. Current Psychiatry Reports.

[10] Hermesh H, Konas S, Shiloh R, Dar R, Marom S, Weizman A, Gross-Isseroff R (2004). Musical hallucinations prevalence in psychotic and nonpsychotic
outpatients. J Clin Psychiatry.

[11] Ukai S, Yamamoto M, Tanaka M, Shinosaki K, Takeda M (2007). Donepezil in the treatment of musical hallucinations. Psychiatry Clin Neurosci.

[12] Tanriverdi N, Sayilgan MA, Ozçürümez G (2001). Musical hallucinations associated with abruptly developed bilateral
loss of hearing. Acta Psychiatr Scand.

[13] Mocellin R, Walterfang M, Velakoulis D (2008). Musical hallucinosis case reports and possible neurobiological
models. Acta Neuropsychiatr.

[14] Roth M, Tym E, Mountjoy CQ (1986). CAMDEX A standardised instrument for the diagnosis of mental disorder
in the elderly with special reference to the early detection of
dementia. Br J Psychiatry.

[15] Folstein MF, Folstein SE, McHugh PR (1975). Mini-Mental State a practical method for grading the cognitive state
of patients for the clinician. J Psychiatr Res.

[16] Brucki SMD, Nitrini R, Caramelli P, Bertolucci PHF, Kamoto I (2003). Sugestões para o Uso do Mini-Exame do Estado Mental no
Brasil. Arq Neuropsiquiatr.

[17] Mendez MF, Ala T, Underwood K (1992). Development of scoring criteria for the clock drawing task in
Alzheimer's disease. J Am Geriatr Soc.

[18] Shulman KI, Gold DP, Cohen CA, Zucchero CA (1993). Clock-drawing and dementia in the community a longitudinal
study. Int J Geriatr Psychiatry.

[19] Yesavage JA, Brink TL, Rose TL (1983). Development and validation of a geriatric depression screening scale a
preliminary report. J Psychiatr Res.

[20] Pfeffer RI, Kurosaki TT, Harrah CH, Chance JM, Filos S (1982). Measurement of functional activities in older adults in the
community. J Gerontol.

[21] Griffiths TD (2000). Musical hallucinosis in acquired deafness Phenomenology and brain
substrate. Brain.

[22] Lishman WA (1998). Organic Psychiatry - The psychological consequences of cerebral
disorders.

[23] Rocha SCM (2012). Uso de prótese auditiva no controle do zumbido e alucinação musical..

[24] Boza RA (1999). Hallucinations and illusions of non-psychiatric
aetiologies. Psychiatri On-line.

[25] Santos RM, Sanchez TG, Bento RF, Lucia MC (2012). Auditory hallucinations in tinnitus patients Emotional relationships
and depression. Int Arch Otorhinolaryngol.

